# XXVI Reunión Anual de la Sociedad Extremeña de Neurología. Comunicaciones Orales y Póster

**DOI:** 10.31083/RN38009

**Published:** 2025-04-28

## Comunicaciones Orales

**1. Actuaciones del Equipo PROA Sobre Pacientes con Patología Neurológica en el Hospital Universitario de Cáceres**

Alberto Barneto Clavijo^1^, Lucía Macarena Olea Ramírez^1^, María Garcés Pellejero^1^, Lorena López Gata^1^, Inés García Gorostiaga^1^, Juan Luengo Álvarez^2^, María Isabel Martín Martín^2^, Ignacio Casado Naranjo^1^

^1^Servicio de Neurología, Hospital Universitario de Cáceres, 10004 Cáceres, España ^2^Servicio de Medicina Interna, Hospital Universitario de Cáceres, 10004 Cáceres, España

**Objetivos**: Determinar la correcta prescripción de antibióticos en pacientes con patología neurológica aguda en situación de hospitalización, tanto en patología infecciosa neurológica como en complicaciones infecciosas y establecer la relación existente entre el servicio de neurología y la unidad PROA/Infecciosas del Hospital Universitario de Cáceres. **Material y Métodos**: Estudio observacional retrospectivo con la inclusión de 23 pacientes hospitalizados en planta de neurología del Hospital Universitario de Cáceres entre marzo y noviembre de 2023 con patología neurológica aguda que presentaron semiología infecciosa neurológica o infección concomitante. Se analizaron variables como sexo, edad, diagnóstico neurológico, diagnóstico infeccioso e intervenciones dirigidas por PROA como desescalada y escalada antibiótica, finalización del tratamiento, tratamiento empírico o terapia secuencial oral. Elaboración de base de datos y analítica descriptiva de resultados con apoyo de programa SPSS. **Resultados**: Durante este período se realizaron 32 actuaciones por parte de la unidad PROA en pacientes hospitalizados con patología neurológica, de los que el 73,9% de los casos se trataba de ictus y el 26,1% de meningitis-encefalitis. En cuanto al diagnóstico infeccioso, en el 34,8% de los casos se trataba de infecciones respiratorias y en el 17,4% de infecciones del tracto urinario. Estas intervenciones fueron tratamiento empírico en el 26% de los casos, tratamientos dirigidos en el 22% y en el 17% de los casos se realizó finalización del tratamiento o desescalada antibiótica con un índice de aceptación de las recomendaciones del 100% por parte de los neurólogos responsables. **Conclusiones**: La colaboración clínica entre el Servicio de Neurología y la Unidad de Infecciosas-PROA de Cáceres se trata de una relación de confianza, con el asesoramiento en la prescripción de terapia antibiótica, en base a la experiencia de los profesionales de la Unidad PROA dada la frecuencia de gérmenes causales y la completa respuesta de los facultativos de neurología a las recomendaciones realizadas, como queda constancia en el 100% de aceptación, en pro de unos mejores resultados en salud de los pacientes atendidos en planta de neurología.

**2**.

**Ecocardioscopia en el Paciente con Ictus Isquémico**

María Garcés Pellejero^1^, Elena Morales Vacas^1^, Lorena López Gata^1^, Lucía Olea Ramírez^1^, Alberto Barneto Clavijo^1^, Inés García Gorostiaga^1^, Juan José Duarte Martín^1^, Alfonso Falcón García^1^, Juan Carlos Portilla Cuenca^1^, Victoriano Romero Cantero^1^, Ignacio Casado Naranjo^1^

^1^Complejo Hospitalario Universitario de Cáceres, 10004 Cáceres, España

**Objetivos**: La ecocardioscopia realizada por neurólogos acreditados en pacientes con ictus isquémico es útil para detectar cardiopatía embolígena como causa subyacente. Esta práctica, cada vez más extendida, surge ante la necesidad de obtener información para iniciar medidas terapéuticas de forma precoz. El objetivo de este estudio es analizar si existe correlación entre los datos obtenidos mediante ecocardioscopia realizada por neurólogos acreditados y el ecocardiograma transtorácico (ETT) convencional. **Material y Métodos**: Estudio unicéntrico, retrospectivo en el que se incluyeron 53 pacientes que ingresaron por ictus isquémico o ataque isquémico transitorio que fueron evaluados mediante ecocardioscopia y ETT. Se realizó un test de concordancia entre ambos estudios analizando las siguientes variables: diámetro aurícula izquierda (AI), diámetro raíz aórtica (RA), fracción de eyección del ventrículo izquierdo (FEVI), valvulopatía mitro-aórtica y derrame pericárdico. **Resultados**: La proporción de concordancia observada entre ambos grupos resultó: 77,36 % para la detección de AI (S 77,78 %, E 76,92 %), 93,1 % para RA (S 50%, E 96,3%), 88% para la FEVI (S 66,67%, E 90,91%), 66% para valvulopatía mitral (S 41,67%, E 86,21%), 73,58% aórtica (S 33,3%, E 94,29%) y 96,15% para derrame pericárdico (S 0%, E 100%). **Conclusiones**: En nuestra experiencia, existe concordancia entre la ecocardioscopia realizada por neurólogos acreditados y el ETT convencional. La ecocardioscopia es una técnica accesible y de alta rentabilidad diagnóstica que nos permite actuaciones terapéuticas precoces en la prevención de nuevos eventos embólicos, así como la estratificación del pronóstico y la reducción de la estancia hospitalaria de los pacientes.

**3**.

**Encefalitis de Rasmussen en el Adulto y Solapamiento Funcional: Un Reto Diagnóstico y Terapéutico**

Andrea Parejo Olivera^1^, Noelia Valverde Mata^1^, Marina Mesa Hernández^1^, Pilar Blanco Ramírez^1^, Soledad Jiménez Arenas^1^, David Ceberino Muñoz^1^, Ana Belén Constantino Silva^1^, M Rosa Querol Pascual^1^, Ángel Aledo Serrano^2^

^1^Servicio de Neurología, Hospital Universitario de Badajoz, 06080 Badajoz, España ^2^Instituto de Neurociencias, Hospital Universitario Vithas La Milagrosa, 28010 Madrid, España

**Objetivos**: La encefalitis de Rasmussen es una encefalopatía progresiva, autoinmune con afectación hemisférica unilateral caracterizada por crisis refractarias, epilepsia parcial continua (EPC) y déficits corticales focales. El diagnóstico se basa en criterios clínicos, radiológicos y electroencefalográficos. En el tratamiento se emplean inmunomoduladores y la hemisferectomía funcional. El objetivo de este trabajo es exponer la dificultad en el manejo de la encefalitis de Rasmussen cuando asocia solapamiento funcional. **Material y Métodos**: Descripción de un caso clínico. **Resultados**: Mujer de 22 años diagnosticada de encefalitis de Rasmussen que ingresa por mal control de crisis. Comenzó a los 12 años con crisis de semiología opérculoinsular derecha refractarias; fue diagnosticada a los 16 años al objetivarse atrofia hemisférica derecha y EPC. Evolutivamente, ha presentado clonías hemifaciales y episodios de alteración de conciencia con movimientos arrítmicos pélvicos. En los últimos meses presenta diariamente temblor en pierna izquierda de horas de duración y desviación cefálica de segundos. En la exploración destaca hemiparesia izquierda leve. Se realiza Electroencefalografía (EEG) con registro de un episodio de desviación cefálica asociado a anomalías frontotemporales derechas y RM de cráneo con dos lesiones corticales derechas. Iniciamos tratamiento con corticoides e inmunoglobulinas con mejoría de las clonías hemifaciales y de la desviación cefálica, pero sin cambios en el resto de crisis. **Conclusión**: La encefalitis de Rasmussen provoca crisis refractarias, pero no existen casos descritos de solapamiento funcional. En este caso las características, la ausencia de correlato electroencefalográfico y la falta de respuesta al tratamiento de algunos de los episodios sugieren que, además de crisis epilépticas, coexisten crisis funcionales.

**4**.

**Estudio descriptivo sobre la actividad laboral en demencia y deterioro Cognitivo en Extremadura**

David Manuel Tena Mora^1^, David Jesús Ceberino Muñoz^1^, María Asunción Pons García^1^, Noelia Valverde Mata^1^, Andrea Parejo Olivera^1^, Marina Mesa Hernández^1^, Pilar Blanco Ramírez^1^, Soledad Jiménez Arenas^1^, Pablo Macias Sedas^1^, Rosa Velicia Mata, María José Gómez Baquero^1^, Ana Belén Constantino Silva^1^, Ana María Roa Montero^1^, Mar Marcos Toledano^1^, Víctor Pérez de Colosía Rama^1^, José María Ramírez Moreno^1^, Rosa Querol Pascual^1^, Patrocinio García Moreno^1^, Sandra Bartolomé Alberca^1^, Ana Garrido García^1^

^1^Servicio de Neurología, Hospital Universitario de Badajoz, 06080 Badajoz, España

**Objetivos**: En 2019, Ministerio Sanidad y Consumo publicó Plan Integral Demencias, vertebrando aspectos eticos, legales, sociales y clínicos. Ninguna literatura científica ha analizado tales aspectos en práctica clínica en este contexto. El propósito del presente estudio, es analizar el ejercicio profesional regional neurología, referente a estos aspectos integrales en deterioro cognitivo y demencia e identificar limitaciones al respecto. **Material y Métodos: **Se invitó via e mail, a socios SEXNE, a cumplimentar anónima y voluntariamente, entre 4–15 Abril, un cuestionario, estructurado en 56 preguntas, que abordaba aspectos asistenciales, docentes, investigación, legales y sociales, ligados al ejercicio profesional neurología, en demencias. **Resultados**: 13 participantes **Resultados por Ámbitos**: (a) Asistencial: 46% usaba siempre/casi siempre tests cribado cognitivo, 15% de evaluación neuropsiquiátrica y 8% evaluación funcional y cuidador. 85% y 51% respectivamente, aplicaban exploración neurológica y sistémica. Un 54% derivan siempre/casi siempre a consultas Neuropsicologia. El 77% proporcionan información sobre terapias y material apoyo. (b) Docencia conocimientos integrales 72% estudiantes y 92% MIR. (c) Investigación: 8% curso máster/tesis. 15% publicó artículos y 31% capítulos/libros. (d) Social: 56% brindan información sobre Asociaciones y 77% sobre recursos sociosanitarios públicos. (e) Legal: 46% proporcionan información legal e incentivan tramitación tales procedimientos. 77% detectan limitaciones asistenciales, 69% en investigación y 92% reconocen necesidad mejoras PIDEX. **Conclusiones**: Uso escaso tests y frecuente exploración. Docencia es habitual e integral. Participación anecdótica en investigación. Información asidua sobre sistemas apoyo sociosanitario y medidas legales. Frecuentes limitaciones asistenciales e investigación. Necesidad mejoras PIDEX.

**5**.

**Experiencia del Tratamiento Endovascular en Fase Aguda en el Ictus Isquémico por Disección Carotídea sin Oclusión Intracraneal**

Lucía Macarena Olea Ramírez^1^, Juan Carlos Portilla Cuenca^1^, Lorena López Gata^1^, María Garces Pellejero^1^, Alberto Barneto Clavijo^1^, Elena Morales Bacas^1^, Montserrat Gómez Gutiérrez^1^, Juan Chaviano Grajera^2^, Sergio Luis Moyano Calvente^2^, Paula Andrea Parra Ramírez^2^, Fernando Alonso Ávalos^2^, Ignacio Casado Naranjo^1^

^1^Servicio de Neurología, Complejo Hospitalario Universitario de Cáceres, 10004 Cáceres, España

^2^Servicio de Radiología Intervencionista, Complejo Hospitalario Universitario de Cáceres, 10004 Cáceres, España

**Objetivos**: A pesar de su baja incidencia general, la disección carotídea es responsable de aproximadamente el 20% de los ictus isquémicos en menores de 45 años, ocasionando trombosis, estenosis u oclusión de la luz arterial. Más allá del tratamiento antitrombótico, debido a los escasos datos disponibles sigue sin existir consenso sobre su tratamiento endovascular, a pesar de demostrarse su seguridad. Nuestro objetivo es describir nuestra experiencia con el tratamiento endovascular en fase aguda en una serie de casos de disección carotídea, sin oclusión intracraneal (LVO). **Material y Métodos**: Estudio descriptivo de serie consecutiva de casos de ictus isquémico tras disección carotídea sin LVO tratados endovascularmente en fase aguda. Analizamos las variables: NIHSS basal y pretratamiento, estudio de neuroimagen, aparición de complicaciones asociadas a deterioro clínico, tratamientos antitrombóticos y evolución clínica (definida por NIHSS al alta). **Resultados**: Incluimos 6 pacientes con edad media de 63.5 (15.1). NIHSS basal 7.5 (6 a 15), NIHSS pretratamiento endovascular 9.5 (5 a 15); con una diferencia de +0.67 (–2.3 a 3), no estadísticamente significativa. Se realizó TC perfusión en el 83,33%. Intervalo de tiempo desde inicio síntomas hasta tratamiento 495’ (de 330 a 600’). Aparecieron complicaciones relevantes en el 16,67%. NIHSS al alta 1 (0 a 1) sobre 5 pacientes; con evolución respecto basal de –6 (de –12.7 a 0.7), p 0.06. Al alta todos mantuvieron doble antiagregación. **Conclusiones**: El tratamiento endovascular en nuestros pacientes es una opción segura y eficaz, con buena mejoría clínica.

**6**.

**Paroxismia Vestibular. Fin a la Dicotomía del Vértigo**

Pilar Blanco Ramírez^1^, Pablo Macias Seda^1^, David Jesús Ceberino Muñoz^1^, Noelia Valverde Mata^1^, Andrea Parejo Olivera^1^, Marina Mesa Hernández^1^, Soledad Jiménez Arenas^1^

^1^Servicio de Neurología, Complejo Hospitalario Universitario de Badajoz, 06010, Badajoz, España

**Objetivos**: La Paroxismia Vestibular (PV) es un síndrome vestibular episódico de baja frecuencia. Los síntomas principales son ataques de vértigo que duran desde segundos a unos pocos minutos y ocurren con o sin síntomas auditivos asociados. Se desencadenan por compresión neurovascular pulsátil directa, principalmente de la AICA, en el nervio vestibulococlear. Esto induce a una lesión segmentaria por presión provocando desmielinización. Presentamos el caso de una paciente con PV en la cual fue imprescindible la realización de RM con secuencia FIESTA para el diagnóstico, tras estudio otológico normal. **Material y métodos:** Descripción de un caso clínico y revisión bibliográfica. **Resultados**: Paciente de 61 años sin antecedentes personales de interés. En seguimiento por Otorrinolaringología por síntomas vestibulares de años de evolución, con estudio otoneurológico normal. Presenta episodios de crisis recurrentes de vértigo espontáneo rotatorio con una duración variable, seguidas de un periodo de síntomas vestibulares. Se solicita RM con secuencia FIESTA, que objetiva cruces vasculares con ambos octavos pares. Los resultados sugieren PV y se inicia tratamiento con bloqueantes de los canales de sodio, con adecuada respuesta clínica. **Conclusión**: La PV es un cuadro de descripción reciente en la literatura otoneurológica, benigno, de fácil tratamiento, pero con gran impacto en la calidad de vida del paciente. Conocer sus criterios diagnósticos resulta fundamental para reconocer este cuadro y poder pensar en su tratamiento específico. El tratamiento quirúrgico implica riesgo de infarto tronco encefálico secundario a vasoespasmo. Por ello se reserva solo para casos con mala respuesta o intolerancia al tratamiento médico.

**7**.

**Síndrome de Miller-Fisher Atípico de Repetición Como Presentación Pseudoictal**

Bernardo Cueli Rincón^1^, Olena Romaskevych Krilvulya^1^, Silvia Moreno Pulido^1^, Marta Martínez Acevedo^1^, Esther González Soltero^1^, María Duque Holguera^1^, Macarena Bejarano Parra^1^, Fernando Castellanos Pinedo^1^

^1^Sección de Neurología, Hospital Virgen del Puerto, 10600 Plasencia, España

**Objetivos**: El síndrome de Miller Fisher (SMF), variante de las polineuropatías inmunomediadas agudas, se caracteriza por la triada de oftalmoparesia, ataxia y arreflexia (salvo presentación atípica), precederse mayoritariamente de un antecedente infeccioso, relacionarse con el antigangliosido GQ1b, con infrecuente recurrencia. Su diagnóstico diferencial inicial implica a otras entidades, enlazando con la complejidad diagnóstica de patologías vasculocerebrales (alrededor del 20–40% finalizarían en pseudoictus). Presentamos un paciente con varios episodios recurrentes de SMF atípico. **Material y Métodos**: Paciente de 62 años, con antecedentes de Meningoencefalitis en la infancia con paresia hemifacial izquierda residual, que presentó en 2020 una oftalmoparesia internuclear izquierda, disartria y una hemihipoestesia izquierda bruscas; dos nuevos episodios bruscos en 2022 clínicamente similares, salvo diferencia en la oftalmoparesia, y presencia dismetría del miembro superior izquierdo y ataxia; dos episodios en 2023, con tetradisestesia distal ascendente subaguda con ataxia e hiporreflexia global (el segundo por recurrencia). Práctica mejoría de los eventos en las semanas posteriores, siendo precedidos de infecciones respiratorias en 2020 y 2022, con respuesta a metilprednisolona e inmunoglobulinas intravenosas (IVIg) en 2022 y 2023. **Resultados**: Se efectuaron exámenes de neuroimagen, neurovasculares y cardiológicos, estudios analíticos amplios en suero y líquido cefalorraquideo (LCR), y paneles genéticos en los cuatro primeros ingresos sin hallazgos relevantes, salvo datos de polineuropatía axonal-desmielinizante sensitivo-motora y positividad para antigangliósidos GD2, GT1a y GQ1b. **Conclusiones**: La identificación precoz esta entidad es difícil, máxime con aparición aguda (pseudoictal) y clínica incompleta o atípica, cuyo correcto tratamiento mejora la recuperación de la misma, pudiendo ésto optimizar el empleo de exámenes complementarios en su proceso diagnóstico.

## Comunicaciones Poster

**1**.

**Diagnóstico Metacrónico de Esclerosis Múltiple y Enfermedad de Parkinson: A Propósito de un Caso**

Soledad Jiménez Arenas^1^, Rosa Querol Pascual^1^, David Ceberino Muñoz^1^, Noelia Valverde Mata^1^, Andrea Parejo Olivera^1^, Marina Mesa Hernández^1^, Pilar Blanco Ramírez^1^

^1^Servicio de Neurología, Hospital Universitario de Badajoz, 06080 Badajoz, España

**Objetivos**: La esclerosis múltiple (EM) es una enfermedad desmielinizante inflamatoria crónica del sistema nervioso central que puede asociarse a diversos trastornos del movimiento. La Enfermedad de Parkinson es una entidad poco común en EM. La asociación puede ser coincidente o causado por EM. Podría hablarse de causalidad en el caso de existencia de placas desmielinizantes que afectaran a ganglios basales y vía nigroestriatal, junto a la respuesta a corticoesteroides. Sin embargo, la ausencia de correlación sugeriría coincidencia. Por otro lado, la posible relación podría explicarse por la evidencia genética. Presentamos un caso, donde planteamos como objetivos: Poner de manifiesto la importancia de la sospecha clínica para diferenciar la sintomatología propia de la EM y la que podría corresponderse a una entidad diferente. Realizar una revisión de los posibles mecanismos que justifiquen la aparición de Enfermedad de Parkinson en pacientes con EM. **Material y Métodos**: Descripción de un caso clínico. **Resultados**: Mujer de 59 años con diagnóstico de EMRR y en tratamiento con Rebif 22 mcg desde agosto de 2016, que, de forma simultánea a la progresión de la enfermedad, comienza en 2018 con temblor inconstante en miembro superior derecho de reposo. Es en 2020 cuando la paciente presenta un empeoramiento clínico. Por un lado, presenta mayor pérdida de fuerza y sensibilidad que implica cambio de tratamiento a Teriflunamida y, por otro lado, un aumento del temblor y rigidez articular en extremidades izquierdas que le impide realizar actividades domésticas. En 2021 se añade rigidez articular axial e incapacidad de realizar movimientos de pinza y alternantes en extremidades izquierdas. En 2022 DATSCAN patológico. Inicio de Sinemet plus tras intolerancia a otros tratamientos en junio del 2023 con gran mejoría. Estudio genético E. Parkinson y Cadasil negativos. **Conclusiones**: El desarrollo completo de un síndrome parkinsoniano asimétrico, la excelente respuesta a levodopa y el DATSCAN congruente con la Enfermedad de Parkinson, indican una relación etiopatogénica coincidente con la Esclerosis Múltiple.

**2**.

**Meningoencefalitis Autoinmune Secundaria a Inmunoterapia**

Marina Mesa Hernández^1^, Noelia Valverde Mata^1^, Andrea Parejo Olivera^1^, Pilar Blanco Ramírez^1^, Soledad Jiménez Arenas^1^

^1^Servicio de Neurología, Hospital Universitario de Badajoz, 06080 Badajoz, España

**Objetivos**: Las encefalitis autoinmunes constituyen un grupo de enfermedades inflamatorias del sistema nervioso central mediadas por anticuerpos contra receptores de neurotransmisores o proteínas de la superficie neuronal. La respuesta autoinmune puede iniciarse por la presencia de un tumor o infección vírica. En muchos casos, los pacientes con meningoencefalitis terminan sin un diagnóstico definitivo lo cual supone un hándicap teniendo en cuenta la alta mortalidad y los déficits irreversibles que conlleva. Nuestro objetivo es poner de manifiesto el diagnóstico de encefalitis asociadas a inmunoterapia. Relevancia de la Inmunoterapia en las enfermedades oncológicas y posibles efectos adversos graves. **Material y métodos**: Descripción de un caso clínico. **Resultados: **Hombre de 75 años con antecedentes personales de Tumor renal estadio IV en tratamiento clínico con doble inmunoterapia (Brazo XL092 + Nivolumab), ingresa por fiebre de 9 días de evolución con clínica neurológica acompañante. Se decide llevar a cabo punción lumbar con hallazgos compatibles con meningitis linfocitaria en líquido cefalorraquídeo. Se realiza estudio etiológico completo y tras descartar causas infecciosas o paraneoplásicas se acepta como causa principal una meningitis autoinmune secundaria a inmunoterapia. **Conclusiones**: La inmunoterapia constituye un elemento terapéutico crucial en el tratamiento de enfermedades oncológicas y aunque el perfil de seguridad es óptimo, conlleva riesgo de desarrollar encefalitis autoinmune con secuelas graves e incluso la muerte, por eso es crucial la detección de los síntomas y un diagnóstico temprano.

**3**.

**No Todo es lo que Parece: Monoparesia del VI par Craneal de Etiología Compresiva de Inicio Agudo en Paciente con Factores de Riesgo Vascular**

María Duque Holguera^1^, Silvia Moreno Pulido^1^, Bernardo Cueli Rincón^1^, Victoriano Romero Cantero^2^, Marta Martínez Acevedo^1^, Olena Romaskevych Kryvulya^1^, Macarena Bejarano Parra^1^, M Esther González Soltero^1^, Fernando Castellanos Pinedo^1^

^1^Sección de Neurología, Hospital Virgen del Puerto, Plasencia, 10600 Plasencia, España^2^Servicio de Neurología, Hospital de Denia, 03700 Dénia, España

**Objetivos**: La paresia del VI par craneal es la neuropatía oculomotora aislada más frecuente, siendo la causa más habitual la isquémica por microangiopatía, aunque puede haber otras causas tanto intraaxiales como extraaxiales. Cuando se trata de lesiones compresivas, lo habitual es que se trate de parálisis combinadas o síndromes complejos, por la proximidad anatómica de los nervios. Nuestro objetivo es comunicar una monoparesia aislada del VI par craneal de etiología compresiva en un paciente con múltiples factores de riesgo vascular. **Material y Métodos**: Descripción de un caso clínico. **Resultados**: Varón de 50 años, hipertenso y diabético, portador de DAI tras taquicardia ventricular sintomática en el contexto de miocarditis en 2017. Acude por diplopía de 10 días de evolución binocular horizontal sin dolor con los movimientos, cefalea u otros síntomas. A la exploración destacaba una paresia del VI par craneal derecho sin otras alteraciones. Se realizó TC de cráneo informado como normal, completándose con angio TC arterial y venoso que no mostró alteraciones vasculares, interpretándose como una paresia del VI par craneal de probable origen isquémico. La paresia no mejoró, por lo que se completó estudio con RMN cerebral que mostró una lesión nodular en cisterna prepontina, en el recorrido anatómico del VI par derecho, compatible con meningioma o schwannoma. **Conclusiones**: A pesar de la baja probabilidad de una causa compresiva en una mononeuropatía aislada aguda en paciente con factores de riesgo vascular, se debe completar estudio de imagen, incluyendo RMN cerebral, fundamentalmente en aquellos en los que no hay mejoría de la paresia.

**4**.

**Paraparesia Espástica: La Importancia de la Sospecha y el Estudio Genético Para un Diagnóstico Correcto**

Noelia Valverde Mata^1^, Andrea Parejo Olivera^1^, Marina Mesa Hernández^1^, María Soledad Jiménez Arenas^1^, Pilar Blanco Ramírez^1^, María Rosa Velicia Mata^1^, David Ceberino Muñoz^1^, Ana Belén Constantino Silva^1^, María Rosa Querol Pascual^1^

^1^Servicio de Neurología, Complejo Hospitalario Universitario de Badajoz, 06010, Badajoz, España

**Objetivos**: La adrenoleucodistrofia ligada al X (X-ALD) es el trastorno peroxisomal más frecuente. Mutaciones del gen ABCD1 alteran el metabolismo de los ácidos grasos saturados de cadena muy larga. Existen distintos fenotipos; la adrenomieloneuropatía (AMN) se caracteriza por una axonopatía distal no inflamatoria de vías descendentes largas de la médula espinal. Se espera que las mujeres portadoras de mutaciones sean asintomáticas; sin embargo, este es un ejemplo de que pueden presentar un fenotipo similar a AMN, con paraparesia espástica progresiva. **Material y Métodos**: Descripción de un caso. **Resultados**: Mujer de 63 años valorada en consultas en 2013 por debilidad en miembro inferior izquierdo y dificultad para caminar. Sin antecedentes personales ni familiares neurológicos. En la exploración destacan reflejos exaltados y Babinski bilateral. Las pruebas complementarias muestran una lesión frontal inespecífica, discopatía degenerativa y siringomielia dorsal; potenciales evocados somestésicos abolidos en miembros inferiores. En 2020, el estudio genético de paraparesia espástica hereditaria mediante secuenciación de nueva generación (NGS) da resultado negativo. En 2023 se realiza el análisis del exoma completo con la detección de una variante, (c.2000A>T) p.His667Leu, probablemente patogénica. Actualmente, pendiente de estudio Sanger de la paciente y familiares que procedan. **Conclusión**: Las mujeres portadoras pueden presentar formas graves de inicio temprano, por lo que es imprescindible la sospecha diagnóstica. Un panel amplio de paraparesias espásticas hereditarias puede ser el primer paso en el diagnóstico, junto con el análisis de la variación del número de copias (VNC). En caso de no ser concluyente, si la clínica es compatible, habría que realizar una secuenciación del exoma completo.

